# Characterizing carbonyl compounds and their sources in Fuzhou ambient air, southeast of China

**DOI:** 10.7717/peerj.10227

**Published:** 2020-11-05

**Authors:** Zhen He, Xin Zhang, Yunfeng Li, Xuefen Zhong, Hong Li, Rui Gao, Jinjuan Li

**Affiliations:** 1 College of Resource and Environment Engineering, Guizhou University, Guiyang, China; 2 State Key Laboratory of Environmental Criteria and Risk Assessment, Chinese Research Academy of Environmental Sciences, Beijing, China; 3 Environment Research Institute, Shandong University, Qingdao, China; 4 Fujian Academy of Environmental Sciences, Fuzhou, China

**Keywords:** Carbonyl compounds, Ozone, Source apportionment, Fuzhou, VOCs

## Abstract

In recent years, ozone (O_3_) concentrations in the southeastern coastal areas of China have shown a gradual upward trend. As precursors and intermediates in the formation of O_3_, carbonyl compounds play key roles in the atmospheric photochemical oxidation cycle. To explore the main pollution characteristics of carbonyl compounds in a typical coastal city in southeast China, ambient samples were collected in Fuzhou (the provincial capital of Fujian province, located on the southeast coast of China) and analyzed using high-performance liquid chromatography with ultraviolet detection. The study was continuously carried out at an urban site (Jinjishan) and a suburban site (Gushan) in Fuzhou from May 8 to 20, 2018. The total concentration of 16 carbonyl compounds at the urban site was 15.45 ± 11.18 ppbv, and the total concentration at the suburban site was 17.57 ± 12.77 ppbv. Formaldehyde (HCHO), acetaldehyde, and acetone were the main species detected in the samples, and acetone had the highest concentration among the species detected. The suburban site had a higher formaldehyde/acetaldehyde ratio and lower acetaldehyde/propionaldehyde ratio than the urban site, implying that biogenic sources potentially contributed to the carbonyl compound concentrations at the suburban site. The results of an observation-based model showed that anthropogenic hydrocarbons promoted HCHO production on May 17 at the urban site. Compared to biogenic emissions, anthropogenic activity is a more important source of carbonyl compounds.

## Introduction

Carbonyl compounds are precursors of O_3_ and secondary organic aerosols, and play key roles in the atmospheric photochemical oxidation cycle. The impact of carbonyl compounds on O_3_ pollution has become a hot topic in the field of atmospheric chemistry research (Finlayson-Pitts & Pitts Jr, 1997; Zhu et al., 2019). These species can control the generation rates and efficiency of hydroxyl (HO_2_) and peroxyalkyl (RO_2_) groups in the atmosphere through their photolysis and reactions with OH radicals. Carbonyls are also important intermediates in the photo-oxidation of volatile organic compounds (VOCs) and important precursors of peroxyacetyl nitrate (PAN) and organic acids, which means they have an important influence on the potential effect of atmospheric oxidation on O_3_ pollution.

Carbonyl compounds undergo oxidation reactions with atmospheric oxidants to affect the equilibrium relationship in the photochemical oxidant cycle. At the same time, they are oxidized to form less volatile organic compounds, which gradually enter the particulate phase through condensation and adsorption processes and then form secondary organic aerosols (Crutzen & Andreae, 1990; Brey & Fischer, 2015). Carbonyls also have a direct negative impact on human health. Most aldehydes and ketones are strongly irritating, and can cause respiratory infections, sensitization, carcinogenesis, and mutation. Most of the unhealthy symptoms induced by carbonyl compounds are irritation to the eyes and lungs (WHO, 2000). Among them, the International Agency for Research on Cancer classifies formaldehyde as the first category of human carcinogens (IARC, 2006), which can cause nasopharyngeal cancer (IARC, 2004), and may also be related to leukemia (Zhang et al., 2009).

Carbonyl compounds are abundant components in ambient air. They are mainly formed by the oxidation of biogenic and anthropogenic hydrocarbons (Zhu et al., 2019), and are also directly emitted from natural and anthropogenic sources (Singh et al., 1995; Wu & Wang, 2015; Xiang et al., 2017; Zarzana et al., 2017). Generally, anthropogenic sources are significant in urban areas. Biological sources of carbonyl compounds are also considered important.

In China, the concentrations of carbonyl compounds in the atmosphere are usually high. For example, the atmospheric concentrations of carbonyl compounds measured in Beijing showed that the environmental level of carbonyl compounds is 3–5 times of that in Hong Kong (Pang & Mu, 2006). The concentration of HCHO in Guangzhou is three times that in Japan and two times that in Hong Kong (Feng et al., 2005; Sin, Wong & Louie, 2001; Tago et al., 2005). The high concentrations of carbonyl compounds may be important contributors to serious atmospheric photochemical pollution in Chinese cities.

Fuzhou is a city with relatively good air quality in China, ranking 6th out of 168 key cities in China for ambient air quality in 2019. However, ozone concentrations show an increasing trend year by year. Fuzhou’s current O_3_ pollution situation is likely to be a brand-new challenge for China to face after PM_2.5_ pollution is controlled. Therefore, the situation in Fuzhou deserves attention. The carbonyl compounds are important precursors of O_3_, and the study of its characteristics is of great significance to the control of O_3_ pollution. As important precursors of O_3_, VOCs and NOx have attracted wide attention (Hong et al., 2019; Wang et al., 2017). However, most research to date has focused on non-methane hydrocarbons and carbonyl compounds have received limited attention. This research focuses on Fuzhou carbonyl compounds to explore their pollution characteristics and their relationship with ozone concentration. In this study, carbonyl compounds in ambient air were investigated using offline 2,4-dinitrophenylhydrazine (DNPH) cartridge sampling with high-performance liquid chromatography (HPLC) analysis. Fuzhou is in the western part of the Taiwan Strait and is subject to weaker winds during the monsoon exchange period in May and is less affected by regional transmission. Sixteen carbonyl compounds were analyzed in samples collected from May 8 to 20, 2018 in Fuzhou.

## Materials & Methods

### Sampling sites

**Figure 1:**
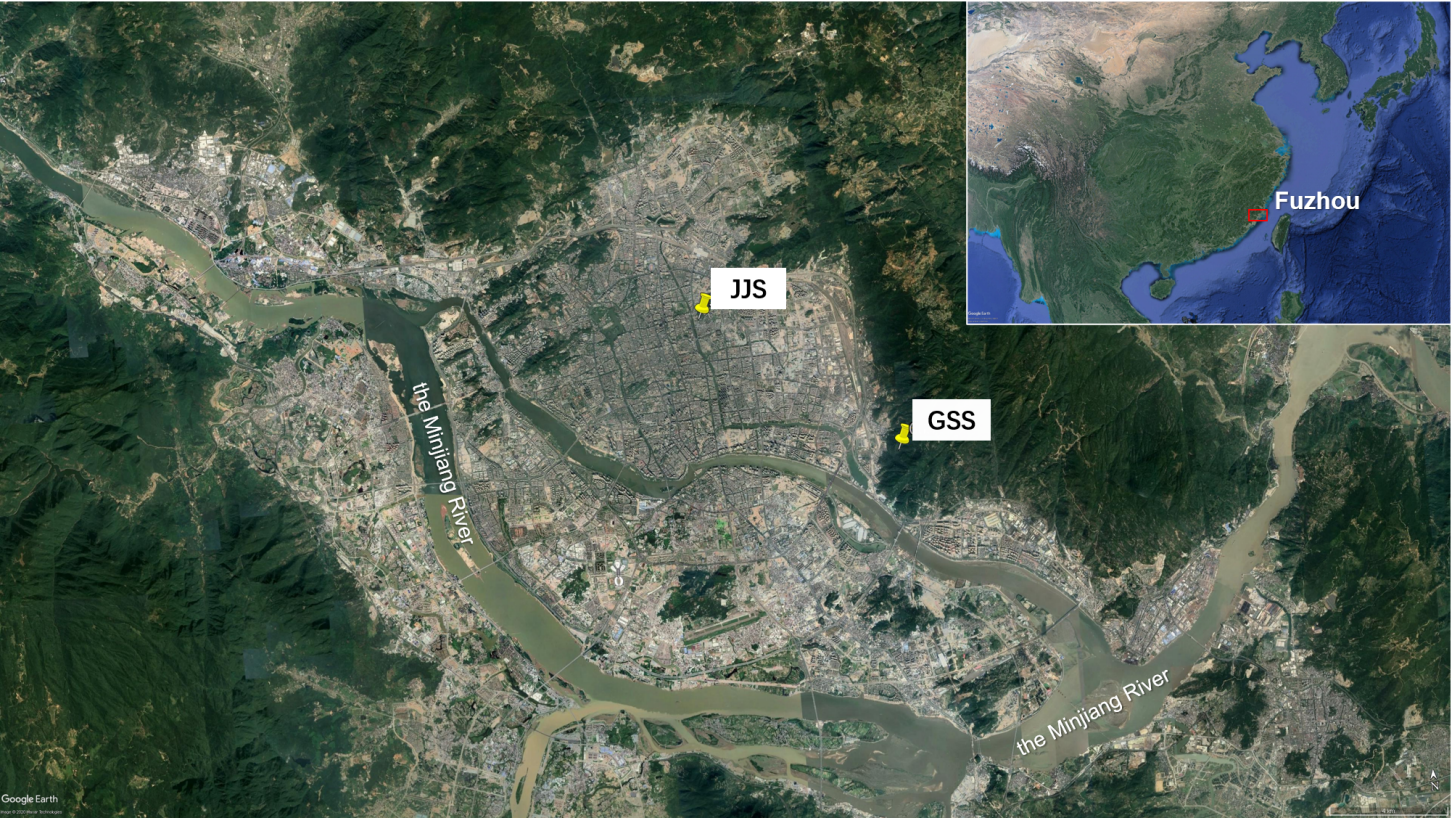
The location of the Fuzhou (Map data ©2020 Google Earth, Date SIO, NOAA, U.S. Navy, NGA, GEBCO, Image Landsat/Copernicus) and the sample sites of JJS and GSS (Map data ©2020 Google Earth, 2020 Maxar Technologies).

Two observation sites were chosen on the rooftop of the Jinjishan Environmental Protection Building (Jijishan site: JJS) and in the Gushan Scenic Area (Gushan site: GSS) in the suburbs of Fuzhou, China (Fig. 1).

Fuzhou is located on the southeast coast of China. The JJS site was located 30 m above the ground at the foot of the southwest Jinjishan mountain in the Jin’an district, Fuzhou. The Jin’an River was located to the west of this site and Jinjishan Park to the northeast. The site was surrounded by residential areas and was considered a typical urban site with no pollution sources. The GSS site was located on the top of the Songtao Building of the Gushan Scenic Area Management Committee. This site was located to the south of the east side of Fuzhou city and north of the Minjiang River. The GSS was selected by China National Environmental Monitoring Center (CNEMC) as a suburban background site as it was surrounded by green trees and had no pollution sources. This site was located about 8 km away from the JJS site.

### Carbonyls sampling and analysis

The sampling and analysis procedures used were based on the Environmental Protection Agency method TO-11A (US EPA, 1999). Ambient carbonyls were collected on silica cartridges coated with acidified DNPH (IC-DN3501, Agela Technologies, China). The sampling flow rate was 0.8 L/min. The sampling pumps in this study are all vacuum pumps with a rated voltage of 24v, and soap film flowmeters are used for flow calibration before sampling (Zhang et al., 2019a). Samples were collected at a 2 h intervals beginning at 00:00 (UTC+8) from May 8 to 20, 2018. The sampling duration was 2 h with 12 atmospheric samples collected each day: 00:00-02:00; 02:00-04:00; 04:00-06:00; 06:00-08:00; 08:00-10:00; 10:00-12:00; 12:00-14:00; 14:00-16:00; 16:00-18:00; 18:00-20:00; 20:00-22:00; 22:00-00:00. A total of 156 carbonyl compound samples were collected at each site. An O_3_ scrubber loaded with potassium iodide (KI 140, Agela Technologies, China) was installed in front of the cartridge to eliminate O_3_. Two cartridges in series were sampled to evaluate the collection efficiency before application to the first sample collection in the field. Over 98% of carbonyls were detected in the first cartridge. After sampling, the cartridges were sealed with Teflon caps immediately, placed in an aluminum foil bag to protect them from light, and stored in a refrigerator (<4 °C). Throughout the sampling process, blank samples were collected at each location. Blank samples were collected by passive sampling, which involved opening a sealed cartridge and placing it in the ambient air at the same time as a cartridge was used for active air sampling. Six cartridges were collected every 12 h, and the concentration of blank samples with no sampling for 12 h had been deducted from the concentration of each sample. Two filed blank samples were collected both before and after the sampling process. At the end of the sample collection period, all the samples were transported to the Laboratory of Atmospheric Photochemical Simulation at the Chinese Research Academy of Environmental Sciences (Beijing, China) in a heat-resistant incubator maintained at 0 °C and then analyzed within 1 month.

In the sampling cartridges, the ambient carbonyls reacted with DNPH to form stable hydrazone derivatives. These derivatives were eluted slowly from the cartridges into a volumetric flask using five mL of acetonitrile (HPLC grade, J.T. Baker, USA). No DNPH or derivatives remained in the cartridges after this elution. Because we several eluted cartridges were chosen to be eluted with 5 ml of acetonitrile again, and no residual carbonyl compounds in the second eluted solutions were detected. This shows that 5 ml of acetonitrile is enough to elute all the carbonyl compounds in the cartridges. The extracts were then analyzed by HPLC (LC20A, Shimadzu, Japan) with UV-Vis detection at 360 nm. The separation column was a Inertsil ODS-P reversed-phase column (250 × 4.6 mm i.d., 5 µm particle size; Shimadzu, Japan) at 40 °C. The mobile phase consisted of acetonitrile and water and the following gradient elution was used: 0–20 min, 60% acetonitrile; 20–30 min, 60% to 100% acetonitrile; 30–32 min, 100% to 60% acetonitrile; and 32–40 min, 60% acetonitrile. The total flow rate was 1.0 mL/min and the injection volume was 20 µL. A mixed calibration standard of 15 DNPH-carbonyl derivatives (Supelco, Bellefonte, PA), which contained HCHO, acetaldehyde, acrolein, acetone, propionaldehyde, crotonaldehyde, butyraldehyde, benzaldehyde, isovaleraldehyde, valeraldehyde, o-tolualdehyde, m-tolualdehyde, p-tolualdehyde, hexaldehyde, and 2,5-dimethylbenzaldehyde, and a calibration standard of methacrylaldehyde (MACR) were diluted to 0.03, 0.06, 0.09, 0.15, 0.24, 0.30, and 0.45 µg/mL. Details for the detection of the 16 carbonyl compounds are given in Table 1.

**Table 1:** Detection information of 16 carbonyl compounds by HPLC/UV.

**Species**	**Retention time (min)**	**Correlation coefficient (R**^**2**^**)**	**Detection limit (ppbv)**	**Quantitation limit(ppbv)**
Formaldehyde	4.97	1.0000	0.022	0.073
Acetaldehyde	6.24	1.0000	0.022	0.073
Acrolein	7.83	1.0000	0.023	0.076
Acetone	8.13	1.0000	0.026	0.085
Propionaldehyde	8.70	1.0000	0.028	0.092
Crotoraldehyde	10.55	1.0000	0.031	0.102
*n*-Butyraldehyde	12.00	0.9998	0.035	0.104
Benzaldehyde	13.31	0.9994	0.036	0.109
Isovaleraldehyde	16.20	0.9996	0.049	0.164
Valeraldehyde	17.13	0.9980	0.048	0.159
*o*-Tolualdehyde	18.32	0.9992	0.050	0.166
*m*-Tolualdehyde	18.95	0.9982	0.050	0.166
*p*-Tolualdehyde	19.76	0.9978	0.054	0.180
Hexaldehyde	24.21	0.9998	0.034	0.114
2,5-Dimethylbenzaldehyde	24.75	0.9998	0.031	0.102
MACR	11.67	0.9994	0.033	0.110

### Other measurements

The O_3_ concentrations were measured by a UV photometric O_3_ analyzer (Model 49i, Thermo Fisher Scientific, USA). The NO and NO_2_ concentrations were measured by a chemiluminescence instrument (Model 42i, Thermo Fisher Scientific) coupled with a molybdenum oxide catalytic converter. The SO_2_ and CO concentrations were measured by a pulsed fluorescence analyzer (Model 43i, Thermo Fisher Scientific) and an infrared absorption analyzer (Model 48i, Thermo Fisher Scientific), respectively. The PM _2.5_ concentrations were measured by a multiangle absorption photometer (Model 5012, Thermo Fisher Scientific). The pollutants data (including O_3_, CO, NO_2_, SO_2_, PM _2.5_) came from the National Environmental Monitoring Station in Fuzhou. All instruments in these sites are maintained by a professional service company every day and are turned on during the carbonyl compound sampling period.

The VOCs concentrations were determined on an O_3_ pollution day (May 17) at the JJS site. Four samples were collected in 3.2-L stainless steel canisters at 8:00, 14:00, 18:00, and 21:00 local time. Fifty-seven VOCs species of Photochemical Assessment Monitoring Stations (PAMS) were identified using a gas chromatograph equipped with a flame ionization detector and a mass spectrometry detector (EPA/600-R-98/161, Technical Assistance Document for Sampling and Analysis of Ozone Precursors) at the same laboratory in Chinese Research Academy of Environmental Sciences. Meteorological data for the temperature, relative humidity, wind speed, wind direction, and pressure were obtained from the National Climate Data Center of the National Oceanic and Atmospheric Administration (National Climatic Data Center, 2019).

### Observation-based model

For quantification of the in situ photochemical production and sensitivity analysis of carbonyl compounds, an observation-based model (OBM) was utilized in this study. This model has been successfully applied in previous studies (He et al., 2019; Mellouki, Wallington & Chen, 2015; Xue et al., 2014; Yang et al., 2018; Zhang et al., 2019a; Zhang et al., 2019b). Briefly, it is built on the Master Chemical Mechanism, which is a near-explicit mechanism describing the oxidation of 143 primary VOCs together with the latest IUPAC inorganic nomenclature (Jenkin et al., 2003; Saunders et al., 2003). In this study, the model was updated to the newest version of the Master Chemical Mechanism (VERSION 3.3.1 Available at http://mcm.leeds.ac.uk/ MCM/ accessed on 3 January 2020). In the calculations, the observed concentrations of O_3_, NO, NO_2_, SO_2_, CO, and VOCs, and the temperature, relative humidity, and pressure were interpolated to a time resolution of 1 h and processed as the model input data sets. We only analyzed the case at JJS station on May 18, 2018 because of the VOCs data limited.

## Results

### Concentration level

**Figure 2:**
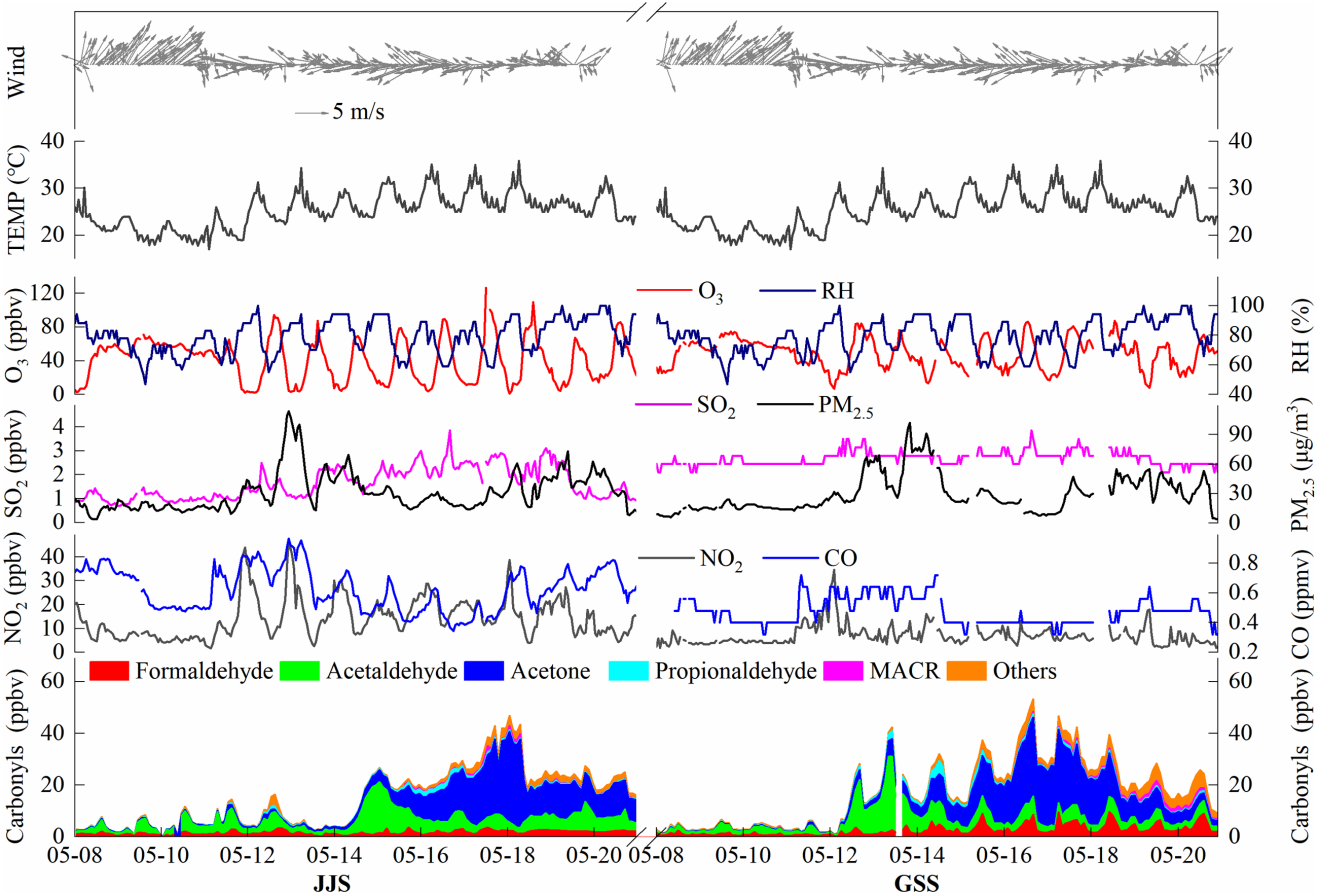
Time series of carbonyl compounds, O_3_, NO_2_, SO_2_, CO, PM_2.5_, and meteorological parameters at GSS and JJS sites from May 5 to 20, 2018.

Figure 2 shows the measured time series of major carbonyl compounds, O_3_, NO_2_, SO_2_, CO, PM_2.5_, and meteorological parameters at the JJS and GSS sites in Fuzhou from May 8 to 20, 2018. The average concentrations of O_3_, NO_2_, SO_2_, CO, and PM_2.5_ during the observation period at the GSS site were 48.29 ppbv, 6.99 ppbv, 2.67 ppbv, 0.47 ppmv, and 29.40 µg/m^3^, respectively. At the JJS site, the corresponding average concentrations were 40.44 ppbv, 14.18 ppbv, 1.63 ppbv, 0.62 ppmv, and 31.06 µg/m^3^ (Table 2).

**Table 2:** Concentrations of PM_2.5_, SO_2_, O_3_, CO, NO_2_ at GSS and JJS sites.

Compound	Mean ± SD		Number of samples	
	GSS	JJS	GSS	JJS
PM_2.5_ (µg/m^3^)	29,40 ± 18.83	31.06 ± 19.36	*n* = 311	*n* = 311
SO_2_ (ppb)	2.67 ± 0.34	1.65 ± 0.66	*n* = 311	*n* = 311
O_3_ (ppb)	48.29 ± 18.03	40.70 ± 25.37	*n* = 311	*n* = 311
CO (ppm)	0.47 ± 0.09	0.63 ± 0.14	*n* = 311	*n* = 311
NO_2_ (ppb)	6.99 ± 4.15	14.22 ± 8.45	*n* = 311	*n* = 311

During the observation period, the concentrations of carbonyl compounds at the JJS and GSS sites were relatively low from May 8 to 11, and the. This may have been caused by low temperatures (average temperature was 21.3 °C) that were not conducive to the formation of carbonyl compounds or strong southwesterly winds that facilitated dispersion of the pollutants. From May 12 to 18, the carbonyl compound concentrations at the two sites greatly increased, which may have been caused by high temperatures (average temperature was 27.1 °C) and strong easterly winds facilitating the formation and accumulation of pollutants. On May 19 and 20, the temperature dropped and the wind direction changed, which resulted in decreases in the concentrations of carbonyl compounds at the two sites. The O_3_ concentration changes were consistent with those for the carbonyl compounds during the observation period. The O_3_ concentration remained at a low level from May 8 to 11, greatly increased from May 12 to 18, and slightly decreased on May 19 and 20. In addition, from May 12 to 20, the GSS site showed large diurnal variations in the concentrations of O_3_ and the carbonyl compounds, and these concentrations peaked at almost the same time (around noon), which indicated that photochemical reactions were the main contributor to O_3_ and carbonyl compound formation during the day at the GSS site. A similar situation was also observed at the JJS site. However, from May 13 to 19 at the JJS site, no obvious daily trends were observed in the carbonyl compound concentrations. These results suggested that anthropogenic sources around the site had considerable contributions in addition to the contribution of photochemical reactions during the day. These observations indicate that the photochemical pollution phenomena occur in Fuzhou.

The overall trends for the carbonyl compounds at the two sites were similar, with a peak in the middle of the day, but the carbonyl compound composition varied greatly with time.

Among the carbonyl compounds detected at the GSS site from May 8 to 11, the dominant carbonyl compound was acetaldehyde, followed by HCHO. From May 12 to 13, the concentration of acetaldehyde increased sharply, and the main reason for this was the overall increase in the concentration of carbonyl compounds. In addition, the concentrations of other carbonyl compounds also increased. From May 14 to 17, the concentration of acetaldehyde decreased and that of acetone increased rapidly. The increasing trend of acetone is due to the conversion of acetaldehyde and the gradual accumulation of acetone. In addition, changes in wind direction and the influence of other man-made pollution sources may be the cause of this phenomenon. The concentration of acetone was much higher than that of any other species. During the observation period, the concentrations of the species varied greatly. The daily concentration changes were basically the same, indicating a stable source of the carbonyl compounds. From May 18 to 20, the total concentration of carbonyl compounds declined as the acetone concentration decreased.

From May 8 to 14, the JJS site was similar to the GSS site and the carbonyl compound concentration was relatively low. Starting on May 14, the concentration of acetaldehyde increased greatly and the concentration of acetone increased slightly, which was the main reason for the large increase in the carbonyl compound concentration. From May 15 to 18, the concentration of acetone increased rapidly and it became the main species, followed by acetaldehyde and HCHO, and the concentrations of the compounds continued to increase. From May 18 to 20, the concentration of acetone began to decrease relative to the previous period (May 15 to 18) but the concentration remained high and it was still the dominant species, followed by acetaldehyde and HCHO.

Table 3 shows the average concentrations and ranges of the 16 carbonyl compounds detected at the GSS and JJS sites in Fuzhou. The total average concentration (± standard deviation) of carbonyl compounds at the GSS site was 17.57 ± 12.77 ppbv and the range was 1.11–53.22 ppbv. The total average concentration of carbonyl compounds at the JJS site was slightly lower than that at the GSS site, which was 15.45 ± 11.18 ppbv with a range of 0.04–46.83 ppbv.

**Table 3:** Concentrations of carbonyl compounds at GSS and JJS sites.

Compound	Mean ± SD^a^ (ppbv)		Range (ppbv)	
	**GSS**	**JJS**	**GSS**	**JJS**
Formaldehyde	2.54 ± 2.09	1.64 ± 0.75	0.23–9.63	0.08–3.68
Acetaldehyde	4.41 ± 4.36	4.84 ± 3.63	0.48–29.07	0.02–19.61
Acrolein	BDL^b^	BDL	BDL	BDL
Acetone	7.45 ± 8.13	6.82 ± 8.11	0.03–30.55	BDL–35.18
Propionaldehyde	0.75 ± 0.82	0.54 ± 0.51	BDL–5.28	BDL–2.20
Crotonaldehyde	0.08 ± 0.08	0.07 ± 0.07	BDL–0.34	BDL–0.29
Butyaldehyde	1.28 ± 1.3	0.80 ± 0.92	BDL–4.57	BDL–3.95
Benzaldehyde	0.05 ± 0.03	BDL	BDL–0.22	BDL–0.15
Isovaleraldehyde	0.48 ± 0.43	0.19 ± 0.53	BDL–2.27	BDL–3.71
Valeraldehyde	BDL	BDL	BDL	BDL–0.20
o-Tolualdehyde	BDL	BDL	BDL	BDL
m-Tolualdehyde	BDL	BDL	BDL–0.05	BDL–0.05
p-Tolualdehyde	BDL	BDL	BDL	BDL
Hexaldehyde	0.06 ± 0.05	0.22 ± 0.34	BDL–0.36	BDL–1.01
2,5-Diemthybenzaldehyde	BDL	BDL	BDL	BDL
MACR	0.52 ± 0.57	0.24 ± 0.42	BDL–2.01	BDL–2.37
Total Carbonyl compounds	17.57 ± 12.77	15.45 ± 11.18	1.11–53.22	0.04–46.83

**Notes.**

a SD means standard deviations in pptv.

b BDL: below detection limit.

### Diurnal variations in the ambient Carbonyl compounds

**Figure 3:**
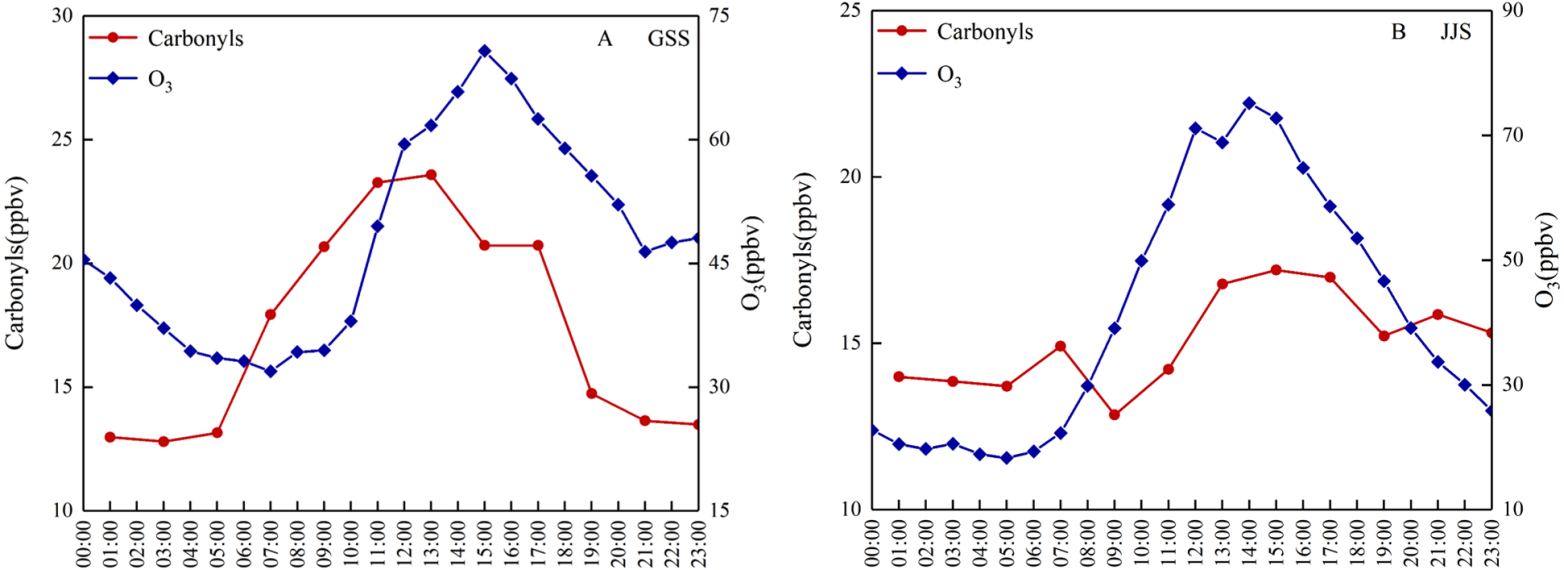
Diurnal variations of the total carbonyl compounds and O_3_ at (A) GSS and (B) JJS sites from May 8 and 20, 2018.

Figure 3 shows diurnal variation of the total carbonyl compound and O_3_ concentrations at the GSS and JJS sites from May 18 to 20, 2018. Both sites showed diurnal variation. It is well-known that atmospheric photochemical reactions are one of the important production routes of carbonyl compounds in ambient air and an important method of O_3_ generation. The intensity of solar radiation directly affects reaction rates in atmospheric photochemistry. Therefore, the intensity of solar radiation has an important influence on the concentrations of carbonyl compounds and O_3_. At the same time, anthropogenic emissions are also important and can affect the concentrations of carbonyl compounds, which directly affect the diurnal variation of carbonyl compounds.

Diurnal variation of the total carbonyl compounds at the GSS site showed a distinct single peak during the observation period. Overall, the total concentration of carbonyl compounds was much higher during the day than at night, with concentrations beginning to increase around 05:00 and peaking at around 12:00–14:00 when solar radiation was the strongest. As solar radiation decreased, the concentration decreased rapidly from 14:00 to 19:00 and stabilized after 19:00. From 19:00 to 05:00 the next day, the concentrations of the carbonyl compounds did not vary much and were maintained at low levels relative to those observed during the day. During the observation period, the daily change in the O_3_ concentration at the GSS site also showed a single peak, and the trend was similar to that observed for the carbonyl compounds.

Diurnal variation at the JJS site was different to that at the GSS site. Generally, variation in the concentrations of carbonyl compounds at the JJS site showed multiple peaks. Similar to the GSS site, the JJS site had higher concentrations of carbonyl compounds during the day than at night; however, the JJS site had much higher concentrations at night (19:00–07:00) than the GSS site, indicating a persistent source of pollution. During the day, the carbonyl compounds showed a peak at 07:00, concentrations then rose from 05:00 to 07:00 before decreasing slightly from 07:00 to 09:00. As solar radiation increased, the concentrations of the carbonyl compounds increased again after 09:00. Between 13:00 and 15:00, the atmospheric photochemical reaction rate reached its daytime peak, and the highest carbonyl compound concentrations were reached at 15:00. From 15:00 to 19:00, as solar radiation decreased, the concentrations of the carbonyl compounds gradually decreased, and between 19:00 to 23:00, a late peak for the carbonyl compounds was reached and then the concentrations began to decrease. The diurnal variation of O_3_ at the JJS site basically showed a single peak that was consistent with the variation observed for the carbonyl compounds from 9:00 to 19:00. This indicated that the concentrations of the carbonyl compounds were mainly affected by atmospheric photochemical reactions during this period.

**Figure 4:**
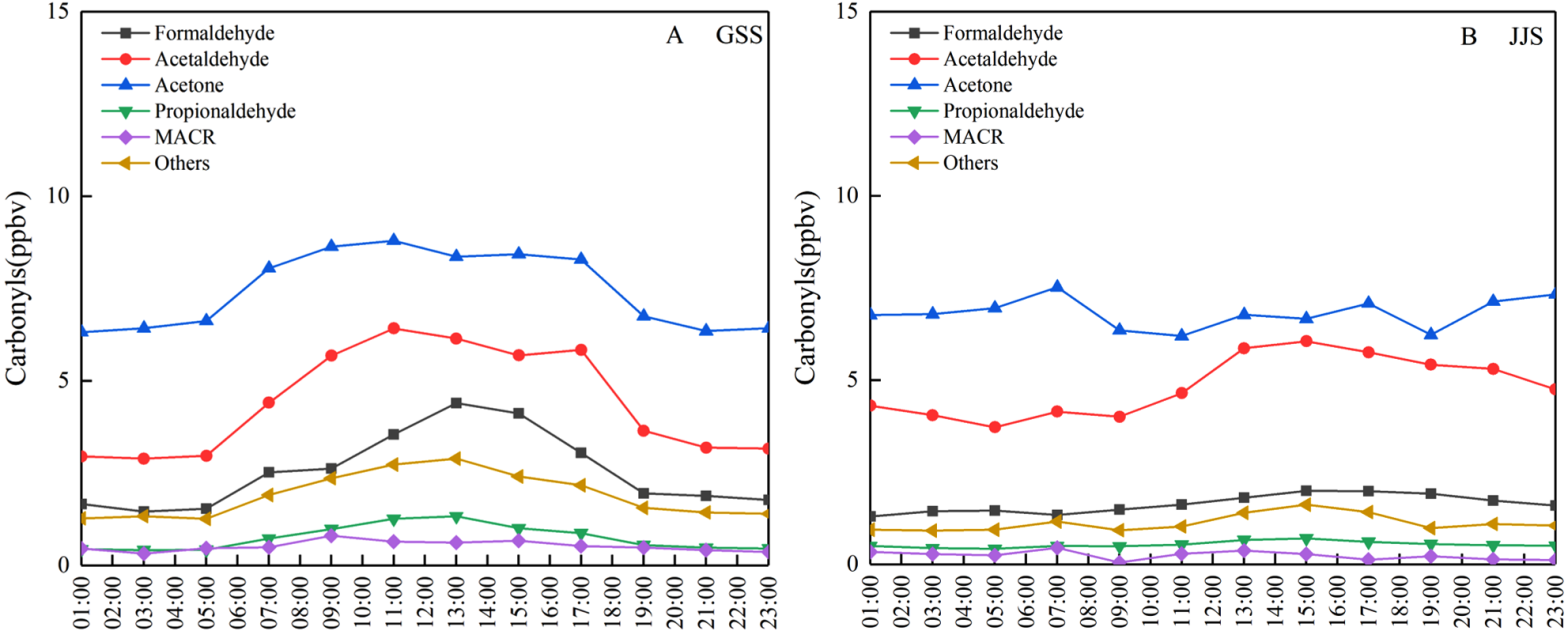
Diurnal variations of main carbonyl compounds at (A) GSS and (B) JJS sites from May 8 and 20, 2018.

Figure 4 shows diurnal variation of the main carbonyl compounds at the two sites from May 8 to 20, 2018. The overall diurnal variation trend for these compounds was similar to that of the total carbonyl compounds. The concentrations of acetaldehyde and acetone at the GSS site were much higher than those of other species, indicating that this was the main species that affected diurnal variation. The diurnal variation of acetaldehyde at the JJS site was consistent with the change in the total carbonyl compounds, indicating that it was one of the main species that affected the total diurnal variation of carbonyl compounds at the JJS site. In addition, the high concentration of acetone also affected the diurnal variation trend of the total carbonyl compounds at the JJS site to a certain extent.

## Discussion

### Comparison of urban and suburban stations

The carbonyl compounds at the GSS site, which had less human activity than the JJS site, mainly arise from secondary generation via photochemical reactions. By contrast, at the JJS site, in addition to secondary generation, human activity in the surrounding area has a high contribution to the carbonyl compound concentrations.

The JJS site was located in an urban area, the pollution sources were more complex than at the GSS site, and the carbonyl compound sources may also be more complex. Although the GSS site was located in a forested area with a single main source of carbonyl compounds, it was close to the JJS site and could be affected pollutants transported from other areas. This may be the main reason why the concentration of carbonyl compounds at the GSS site was slightly higher than that at the JJS site.

**Figure 5:**
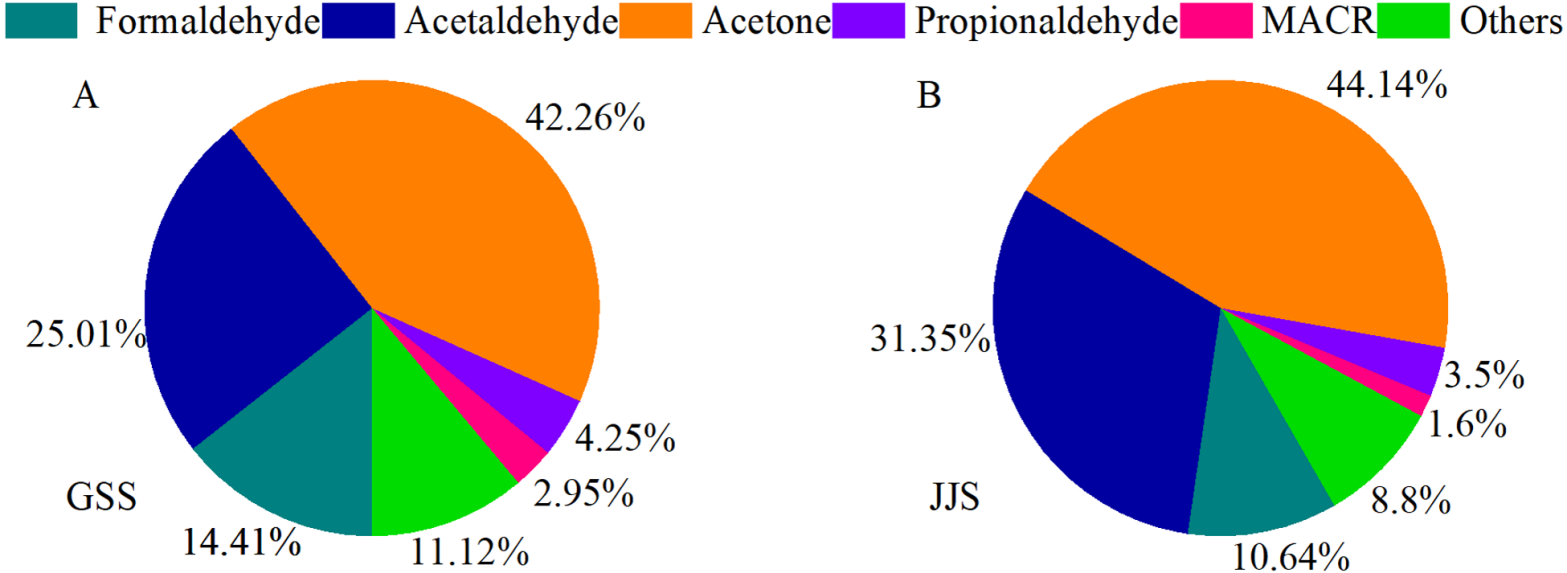
Proportions of main carbonyl compounds at GSS (A) and JJS (B) sites from May 8 to 20, 2018.

Figure 5 shows the average contributions of different carbonyl compounds at the two sites. Acetone was the most abundant carbonyl compound at the GSS and JJS sites, which could be attributed to its chemical stability and long atmospheric lifetime. Acetone released from various sources accumulates in the atmosphere (Atkinson, 2000; Dai et al., 2012) , and the most important sources are natural sources (Janson & Serves, 2001), with pine trees accounting for a higher share of emissions. Among the anthropogenic sources, motor vehicle exhaust emissions are more significant (Janson & Serves, 2001). The average concentration of acetone at the GSS site was 7.45 ± 8.13 ppbv (Table 3), accounting for 42.26% of the total concentration of the 16 carbonyl compounds. The average acetone concentration at the JJS site (6.82 ± 8.11 ppbv) (Table 3) was slightly lower than that at the GSS site, accounting for 44.14% of the total concentration of the 16 carbonyl compounds. These results were comparable to the acetone concentrations measured by Yang et al. (2018). in Beijing. They were also similar to the concentrations measured by Lü et al. (2010) in Guangzhou, where acetone was one of the species with the highest concentrations. The compound with the second highest concentration at the GSS site was acetaldehyde (4.41 ± 4.36 ppbv, 25.01% of the total) (Table 3), and the third was HCHO (2.54 ± 2.09 ppbv, 14.41% of the total) (Table 3). Similar to the GSS site, the species with second highest concentration at the JJS site was acetaldehyde (4.84 ± 3.63 ppbv, 31.35% of the total), and the third was HCHO oxidation (1.64 ± 0.75 ppbv, 10.64% of the total) (Table 3). Higher concentration of MACR could be caused by the abundant vegetation at the GSS site, which would produce more MACR than at the JJS site (Duane et al., 2002; Riemer et al., 1998).

### Diurnal variation analysis

The concentration of carbonyl compounds at GSS sites has an obvious single-peak diurnal variation trend, which is basically consistent with the change of solar radiation intensity during a day, indicating that the concentration of carbonyl compounds is mainly caused by atmospheric photochemical reactions. However, there is no significant diurnal variation in the concentration of carbonyl compounds from the whole-time variation at the JSS site, indicating that the concentration of carbonyl compounds is not only influenced by atmospheric photochemical reactions, but also by certain anthropogenic factors.

The highest concentrations of acetone could be observed at the two sites at any time. Atmospheric oxidation affects the atmospheric lifetime of carbonyl compounds. The photolysis reactions of HCHO and acetaldehyde take about 4 h and 6 days, respectively, and their lifetimes initiated by OH radicals are about 1.2 days (HCHO) and 8.8 h (acetaldehyde) (Dai et al., 2012). Acetone is more stable and has a longer lifetime (60 days uptake by photolysis and 53 days uptake by reaction with OH radicals) than those of HCHO and acetaldehyde. Therefore, acetone accumulates in the atmosphere easily and its concentration is higher than those of other carbonyl compounds such as HCHO and acetaldehyde (Atkinson, 2000; Possanzini, Tagliacozzo & Cecinato, 2007). This further explains why the acetone concentration is much higher than other carbonyl compounds.

### Source apportionment of carbonyl compounds using ratio methods

**Figure 6:**
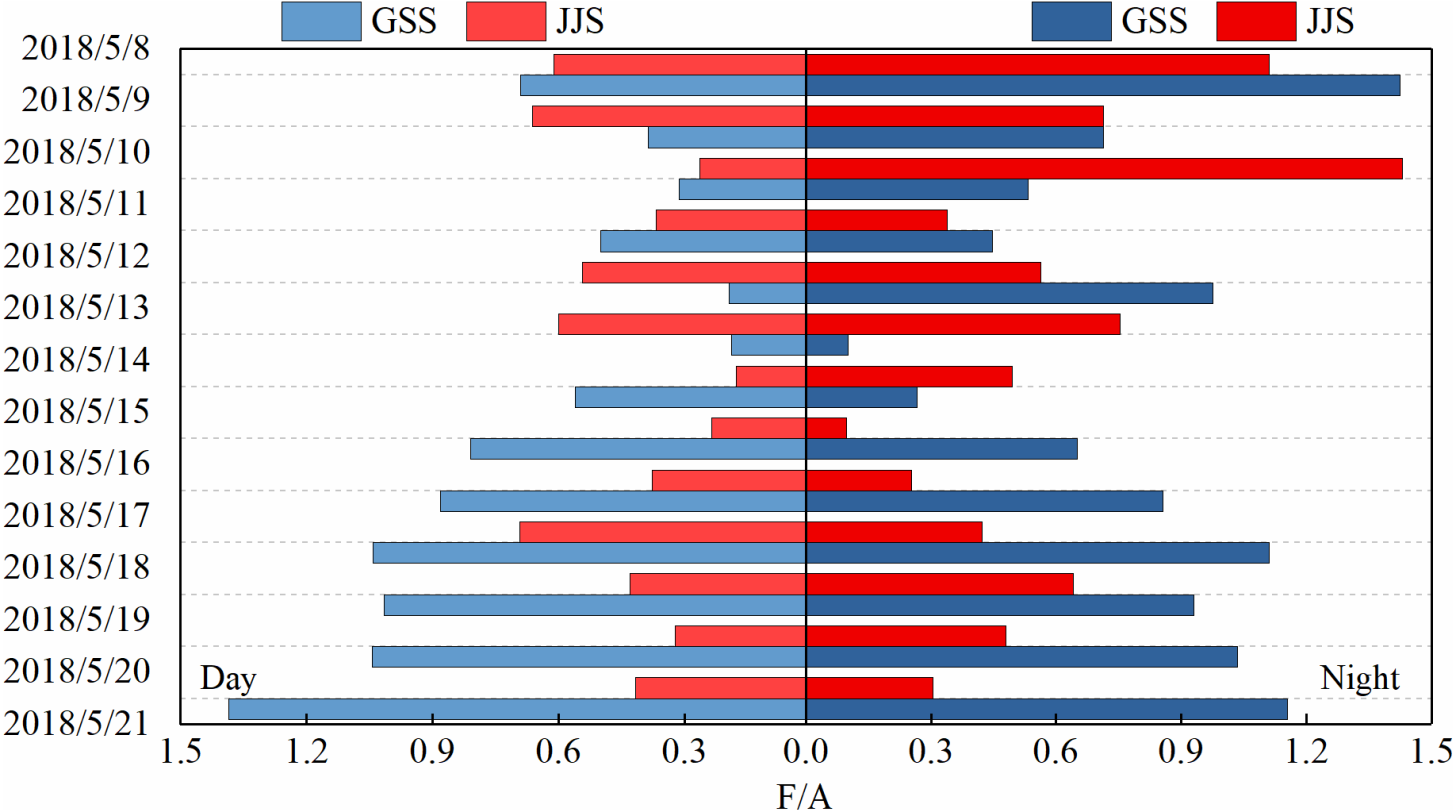
Ratios of formaldehyde to acetaldehyde (F/A) during the day and night at GSS and JJS sites from May 8 and 20, 2018.

**Figure 7:**
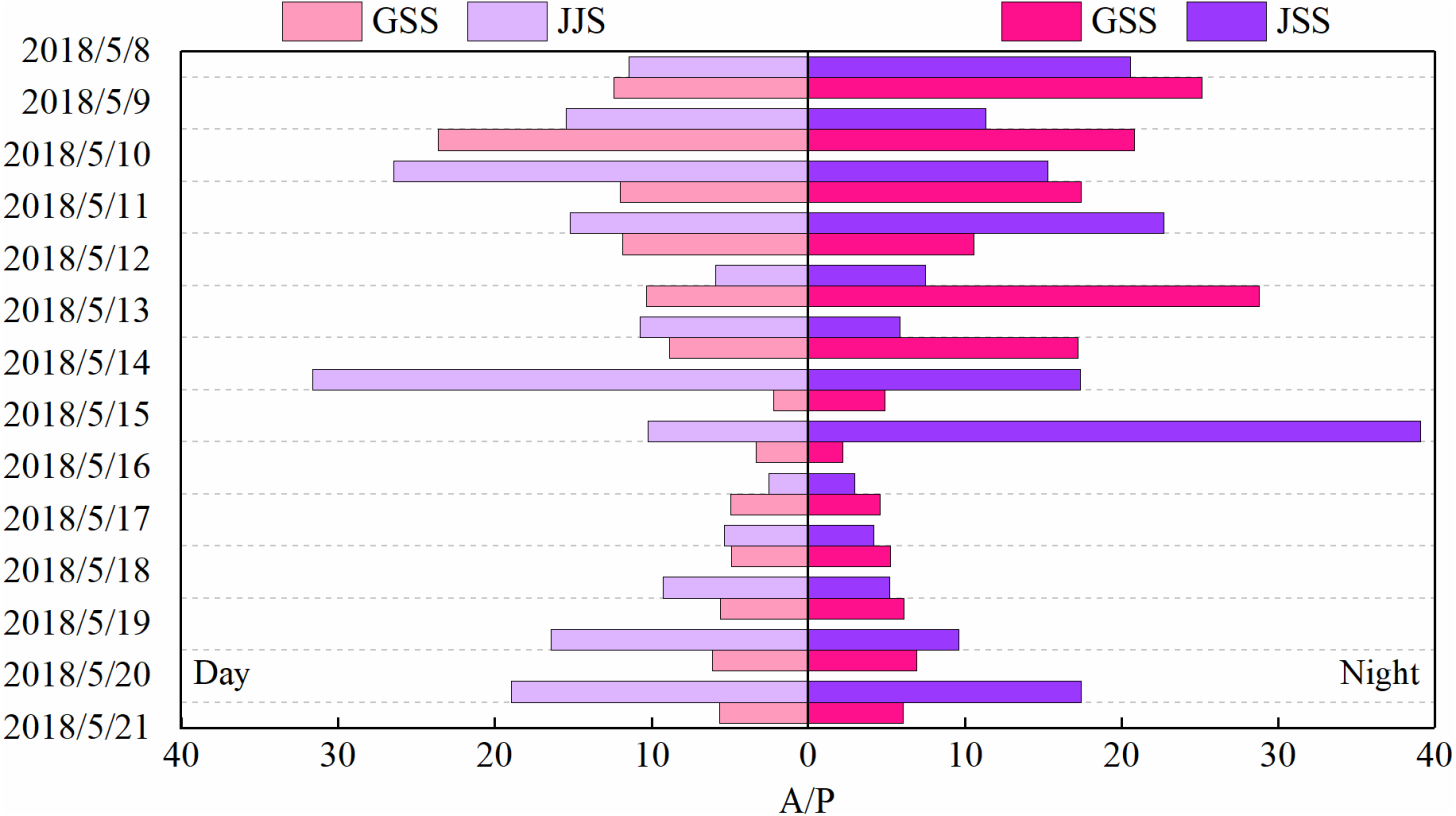
Ratios of acetaldehyde to propionaldehyde (A/P) during the day and night at GSS and JJS sites from May 8 and 20, 2018.

The sources of carbonyl compounds can be roughly determined from the ratio of the concentrations of atmospheric formaldehyde/acetaldehyde (F/A) to that of acetaldehyde/propionaldehyde (A/P) (Shepson et al., 1991). Figures 6 and 7 show statistical analysis of the F/A and A/P ratios during the day and night at the GSS and the JJS sites from May 8 to 21.

The F/A value in the atmosphere is generally between 1–10. Generally, the amount of formaldehyde produced by biogenic-derived hydrocarbons through atmospheric photochemical reactions is higher than the amount of acetaldehyde. Therefore, the F/A ratio is higher in areas with high forest or vegetation coverage than in urban areas (Possanzini et al., 1996; Shepson et al., 1991). Overall, the F/A value at the GSS site (daytime: 0.19–1.38, nighttime: 0.10–1.42) first decreased and then increased during the observation period. The GSS site was located in a forested area and there was one main source of carbonyl compounds. However, the F/A values at the GSS site were much lower than those in typical forested areas, which may be related to the regional transportation of pollutants. During the observation period, the F/A value at the JJS site (daytime: 0.18–0.69, nighttime: 0.10–1.43) also first decreased and then increased.

Overall, the F/A value at the GSS site was slightly higher than that at the JJS site during the observation period. This may be because the vegetation coverage at the GSS site was higher than that at the JJS site as the GSS site was in a forested area. Therefore, the F/A value at the GSS site was higher than that at the urban JJS site. However, because the GSS site was located close to an urban area, the F/A value was lower than that in typical forested area because of the impact of pollutant transportation. The F/A values were slightly higher at night than during the day at both sites, which could be attributed to the fact that formaldehyde and acetaldehyde were removed at night mainly by reactions with NO_3_ radicals. Acetaldehyde reacted at a higher rate than formaldehyde at night. From May 12 to 13, a sudden increase in the GSS acetaldehyde concentration led to a decrease in its F/A value to slightly lower than that at the JJS site.

Generally, the A/P ratio can be used to indicate the presence of man-made pollutants because propionaldehyde is considered only to be associated with anthropogenic sources (Shepson et al., 1991). Therefore, the lower the A/P value, the greater the influence of anthropogenic sources. The A/P at the GSS website was between 2.22–23.64 during the day and 2.20–28.77 at night, while at the JJS site, the A/P was between 2.52–31.62 during the day and 2.99–39.09 at night. Propionaldehyde is mainly related to anthropogenic emissions, and acetaldehyde may come from secondary generation or primary emission. The A/P value at the JJS site was higher than the A/P value at the GSS site. From the A/P ratio, the GSS site seems to be more severely affected by man-made sources, which may be due to the GSS site producing more acetaldehyde. And the GSS site was affected by pollutant transportation from the source area (Table 3), and the concentration of propionaldehyde was slightly higher than that at the JJS site.

The ratio of the concentrations of atmospheric formaldehyde/acetaldehyde (F/A) and acetaldehyde/propionaldehyde (A/P) can be analyzed the relative contribution of anthropogenic and biogenic sources. However, there were some arguments that the ratios of F/A and A/P often have large variations due to different sources of pollution and meteorological conditions (Grosjean, 1992; Ho et al., 2015), thus we should use it with caution. Furthermore, the ratio method also fails to identify different photochemical production. Thus we will discuss the sources of gaseous carbonyls more detail in the next section.

### Source apportionment of carbonyl compounds using observation- based model

**Figure 8:**
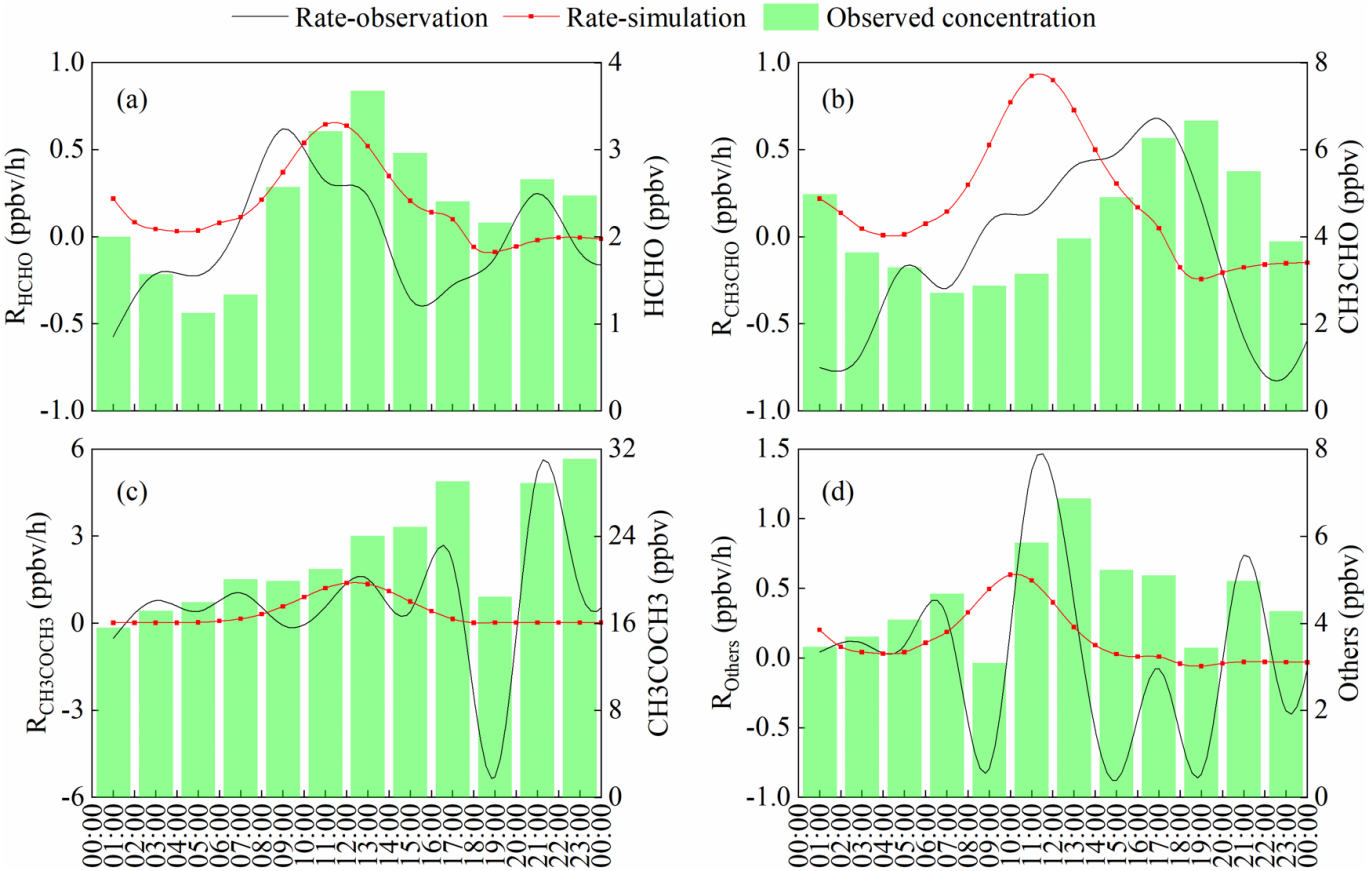
The simulated and observed carbonyls production rate on May 17, 2018. (B) formaldehyde, (B) acetaldehyde, (C) acetone, (D) other carbonyl compounds.

Carbonyls production at the JJS site was observed on May 17, 2018 and simulated by an OBM model (Fig. 8). The simulated distribution of HCHO from photochemical production was compared with the observed HCHO concentration. Peaks appeared during the day and valleys appeared during the night. This was similar to the trend observed for O_3_, and indicated that the HCHO photochemical reaction had a large contribution during the day. The highest in situ HCHO was 0.64 ppbv/h at 12:00, and this value was lower than those measured in a previous study in Beijing on July 23 and 24, 2008 (Yang et al., 2018). One aspect of the figure is of interest, two large increases were observed in HCHO in the early morning and after dusk. Rush hour traffic occurs during these two periods and vehicle emissions are a major source of HCHO at such times (Cao et al., 2016; Tsai, Huang & Chiang, 2014). In addition, the nocturnal boundary layer build ups rapidly after sunset, and unfavorable dilution conditions may contribute to increases in the HCHO concentration (Brown & Stutz, 2012).

The highest observed rate of acetaldehyde appeared at dusk, because the major source of acetaldehyde is anthropogenic emission and it accumulated during daytime. But the highest in situ photochemical rate was 0.92 ppbv/h at 11:00 due to higher photolysis rate of acetaldehyde in the afternoon. Acetone has a lower in situ photochemical rate indicated photochemical generation is the minor source of acetone. And it has a higher background value (the lowest value of acetone, about 16 ppbv) , means it may be affected by local pollution sources on this day. Others represent the sum of other carbonyl compounds. Similar to formaldehyde, it affected by photochemical production during the daytime and anthropogenic emission during the nighttime.

**Figure 9:**
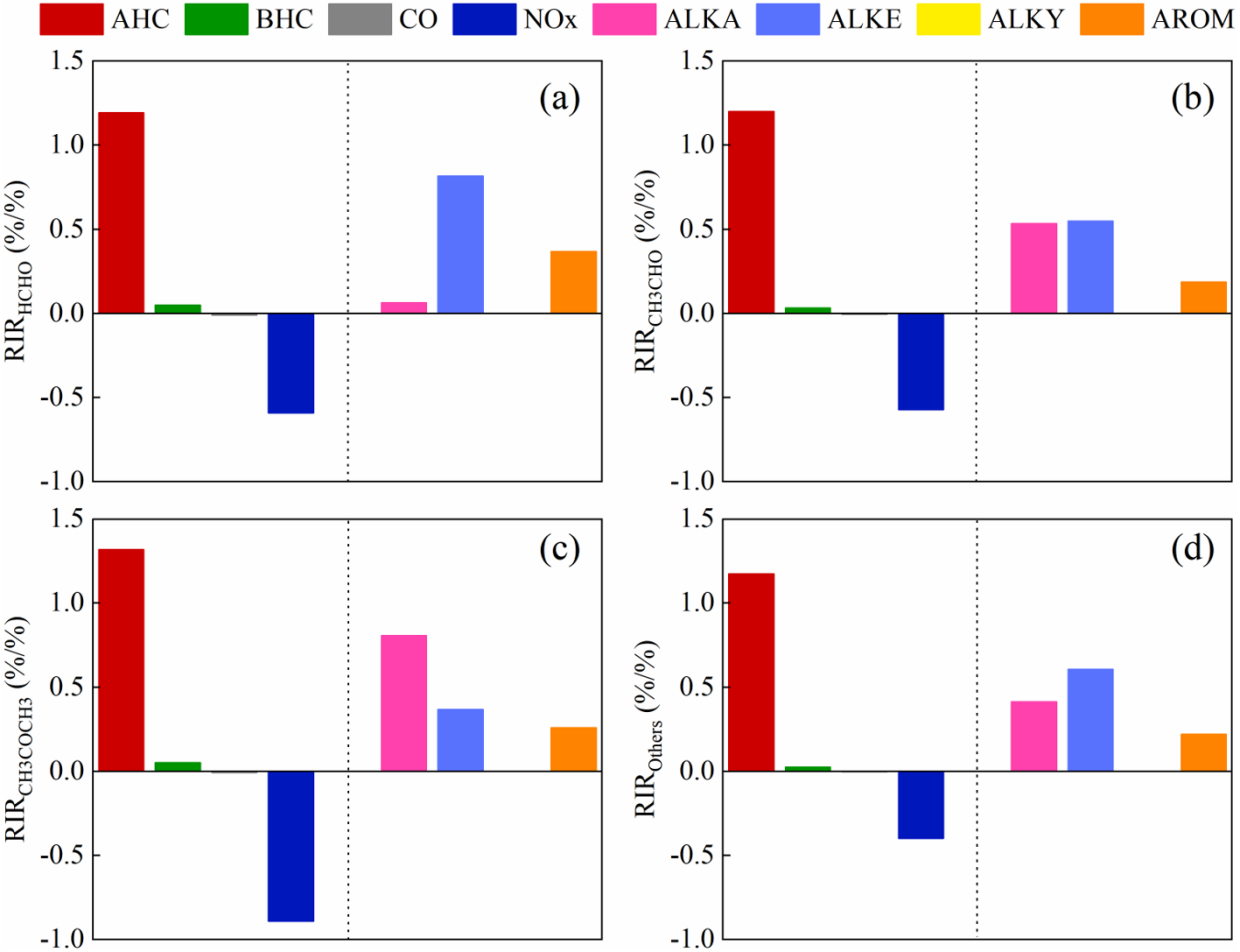
The model-calculated RIR of the major precursors for the secondary formation of carbonyls on May 17, 2018. (A) formaldehyde, (B) acetaldehyde, (C) acetone, (D) other carbonyl compounds.

At 3:00 p.m., the net HCHO generation rate showed a decreasing trend, indicating that the on-site HCHO generation rate was lower than the HCHO destruction rate. In fact, many simulations showed that the concentration of OH radicals was highest in the afternoon during the day (Tan et al., 2019; Zong et al., 2018). The photolysis of formaldehyde is an important source of OH radicals. Negative values occur when the rate of formaldehyde photolysis is greater than the sum of the production rate and the primary release. It has been reported in many articles that the concentration of formaldehyde decreases between 14:00 and 18:00, and according to the published literature, this phenomenon is most pronounced in summer (De Blas et al., 2018; Jiang et al., 2016; Jiang et al., 2019; Yang et al., 2019).

Figure 9 shows that the model-calculated relative incremental reactivities (RIR) of the major precursors for secondary formation of carbonyl compounds on May 17, 2018. We further identified key precursors by calculating the RIR (Cardelino & Chameides, 1995), which has been applied in many previous studies (Tan et al., 2018; Yang et al., 2018). All VOCs species were categorized as biogenic or anthropogenic hydrocarbons (AHCs). The AHCs were divided into the following four subgroups: alkanes, alkenes, alkynes, and aromatics. All kinds of carbonyl compounds production were VOC-limited, and the dominant position was AHC. The RIRs for the NOx were negative. But the dominant species of different carbonyl compounds were different. Alkenes were dominant and aromatics followed for formaldehyde. For acetaldehyde and other carbonyl compounds, alkanes and alkenes both were important for its chemical generation, followed by aromatics. And alkanes dominate the formation of acetone. Therefore, anthropogenic VOCs emissions should be reduced for carbonyl compounds controlling.

## Conclusions

The characteristics and sources of 16 carbonyls compounds were measured in May 2018 in the southeastern coastal city of Fuzhou, China. The concentration at the urban site was 15.45 ± 11.18 ppbv and the concentration at the suburban site was 17.57 ± 12.77 ppbv. HCHO, acetaldehyde, and acetone were the main species in Fuzhou city, and acetone had the highest concentration. The F/A and A/P ratios were used to determine the sources of the carbonyl compounds. Suburban areas with high vegetation coverage had high F/A values, whereas urban areas were greatly affected by human activities and the A/P values were higher. HCHO production was VOC-limited, and AHCs were dominant and particularly sensitive to reactive alkenes. The RIR for NOx were negative and NOx would generally have a negative influence on attempts to control HCHO production. In summary, both anthropogenic emission and biogenic emissions sources were important source of OVOCs in Fuzhou, and the impact of anthropogenic emission was greater, deserve more attention in the future control of ozone precursors, both in Fuzhou and other cities of China.

##  Supplemental Information

10.7717/peerj.10227/supp-1 Supplemental Information 1 Meteorological factors (A), concentration of PM2.5,PM10,SO2,O3,CO,NO2 (B), and concentraion of carbonyl compounds during May 8 to 20, 2018 in Fuzhou

## Additional Information and Declarations

### Data Availability

The following information was supplied regarding data availability: The raw measurements are available as a Supplemental File.
